# Bioinformatic Analysis of Two TOR (Target of Rapamycin)-Like Proteins Encoded by *Entamoeba histolytica* Revealed Structural Similarities with Functional Homologs

**DOI:** 10.3390/genes12081139

**Published:** 2021-07-28

**Authors:** Patricia L. A. Muñoz-Muñoz, Rosa E. Mares-Alejandre, Samuel G. Meléndez-López, Marco A. Ramos-Ibarra

**Affiliations:** Biotechnology and Biosciences Research Group, Faculty of Chemical Sciences and Engineering, Autonomous University of Baja California, Tijuana 22390, Mexico; lilian.munoz.munoz@uabc.edu.mx (P.L.A.M.-M.); rmares@uabc.edu.mx (R.E.M.-A.); samuelmelendez@uabc.edu.mx (S.G.M.-L.)

**Keywords:** target of rapamycin, FKBP-rapamycin associated protein, homology-based protein modeling, structure-function biocomputational analysis, *Entamoeba histolytica*

## Abstract

The target of rapamycin (TOR), also known as FKBP-rapamycin associated protein (FRAP), is a protein kinase belonging to the PIKK (phosphatidylinositol 3-kinase (PI3K)-related kinases) family. TOR kinases are involved in several signaling pathways that control cell growth and proliferation. *Entamoeba histolytica*, the protozoan parasite that causes human amoebiasis, contains two genes encoding TOR-like proteins: *Eh*FRAP and *Eh*TOR2. To assess their potential as drug targets to control the cell proliferation of *E. histolytica*, we studied the structural features of *Eh*FRAP and *Eh*TOR2 using a biocomputational approach. The overall results confirmed that both TOR amoebic homologs share structural similarities with functional TOR kinases, and show inherent abilities to form TORC complexes and participate in protein-protein interaction networks. To our knowledge, this study represents the first in silico characterization of the structure-function relationships of *Eh*FRAP and *Eh*TOR2.

## 1. Introduction

The target of rapamycin, TOR (also known as FKBP-rapamycin associated protein, FRAP), is a conserved Ser/Thr protein kinase that belongs to the eukaryotic PIKK family (phosphatidylinositol 3-kinase (PI3K)-related kinases) [1]. The yeast proteins TOR1p and TOR2p were first identified in *Saccharomyces cerevisiae* as point mutations that conferred a resistance to rapamycin [2,3], an antifungal antibiotic produced by *Streptomyces hygroscopicus* [4,5]. Rapamycin binds to the FK506-binding protein (FKBP) and interacts with TOR proteins through the FKBP-rapamycin binding (FRB) domain [6,7]. Affinity studies showed that the FKBP-rapamycin complex binds more tightly to the FRB domain than rapamycin alone (about 2000-fold), suggesting that the rapamycin-TOR interactions in the absence of FKBP would have minimal effects under physiological conditions. These studies also indicated that protein-protein interactions are important for the FKBP-rapamycin-TOR complex stability [8]. Remarkably, this ternary complex inhibits TOR functions by blocking its structural capability to form TORC1 or TORC2, the multiprotein complexes involved in several cell functions [9,10].

The biochemical analysis of the yeast protein complexes showed that TORC1 contains either TOR1p or TOR2p, whereas TORC2 specifically contains TOR2p [11,12]. Further characterization provided the basis for a functional definition of two signaling branches [13]. TORC1 (formed by TOR1p/TOR2p, Lst8p, Kog1p, and Tco89p) is rapamycin-sensitive and regulates the transcription apparatus, translation machinery, and growth-related processes in response to amino acids and growth factor signals. Conversely, TORC2 (formed by TOR2p, Lst8p, Avo1p, Avo2p, Avo3p, and Bit61p) is rapamycin-insensitive and regulates cell integrity and actin organization throughout the cell cycle [11,13,14,15,16].

Unlike yeast, mammalian cells have a single TOR kinase, called mTOR, which acts as a catalytic subunit in both mTORC1 and mTORC2 [10,17]. Each complex combines mTOR with various proteins and participates in diverse cell functions mediating the signaling induced by nutrients and growth factors. Briefly, mTORC1 (which includes mTOR, Raptor, PRAS40, Deptor, mLst8, Tti1, and Tel2) exhibits a dual function: (1) it promotes anabolic processes stimulating the synthesis of proteins, lipids, and nucleotides; and (2) it inhibits catabolic processes, such as lysosome biogenesis and autophagy. In contrast, mTORC2 (consisting of mTOR, Rictor, mSin1, Protor-(1/2), Deptor, mLst8, Tti1, and Tel2) controls cell survival, cytoskeleton organization, lipogenesis, and gluconeogenesis [10,17,18,19].

**Figure 1:**
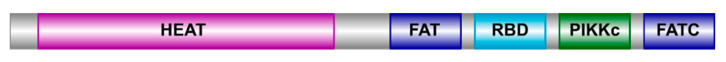
Schematic representation of the canonical domain organization of TOR proteins. HEAT repeats (purple), FAT (dark blue), RBD (light blue), PIKKc (green), and FATC (dark blue). Drawing generated with the IBS program [23].

TOR kinases are multi-domain polypeptides that comprise a canonical organization (Figure 1): a long N-terminal domain including numerous tandem HEAT repeats (Huntingtin, EF3, A-subunit of PP2A, and TOR), which may adopt an extended superhelical conformation; a FAT (FRAP, ATM, and TRRAP) domain; a rapamycin-binding domain (RBD), also known as FRB domain; a PIKKc (phosphatidylinositol 3-kinase (PI3K)-related kinase catalytic) domain; and a FATC (FAT C-terminal) domain [20,21,22].

*Entamoeba histolytica*, an intestinal protozoan parasite, is the causative agent of human amoebiasis, an infectious disease that can lead to colitis, dysentery, and liver abscess [24]. This parasitic disease is also a leading cause of severe diarrhea [24,25,26], which in turn remains among the top 10 causes of death worldwide (https://www.who.int/data/global-health-estimates, accessed on 9 December 2020). Unfortunately, the number of drugs available to treat amoebiasis and thus prevent the spread of *E. histolytica* is limited, with nitroimidazoles (e.g., metronidazole) being the most effective therapeutic option [27]. However, the potential toxicity associated with these drugs and concerns about the emergence of drug resistance [25,27,28], as already reported for other protozoan pathogens [29,30], have encouraged the research and development of new anti-amoebic agents capable of counteracting both intestinal and invasive infections.

Interestingly, rapamycin and other mTOR inhibitors have shown potential as anticancer drugs [31,32,33,34,35], suggesting that TOR kinases may be suitable targets to control cell proliferation [17,31,36,37]. Given their evolutionary conservation [38,39], we used the annotated genomic data of two TOR-like amoebic proteins (*Eh*FRAP and *Eh*TOR2, from now on referred to as such) to further study their structural features through a computational approach, and thus assess their potential as targets for the therapeutic control of human amoebiasis. Overall, results confirmed that both proteins display canonical domain organization and demonstrated that their putative functional domains depict the three-dimensional (3D) structure shown by active TOR kinases.

## 2. Materials and Methods

### 2.1. Sequence Retrieval and Primary Structure Analysis

The sequences of both TOR-like amoebic proteins, *Eh*FRAP (Gene ID: EHI_155160) and *Eh*TOR2 (Gene ID: EHI_169320), were retrieved from the AmoebaDB repository [40]. Their physical-chemical parameters were determined using the ProtParam tool [41]. The orthologous TOR proteins were identified by primary structure comparative analysis using the NCBI BLAST web tool [42]. Next, the putative functional, conserved domains were detected using the NCBI CD-Search engine [43,44], and the protein architecture (i.e., domain organization) was examined using the Pfam Sequence Search engine [45]. As a complementary analysis, the polypeptide repeats were identified, delimited, or confirmed, using the REP2 server [46] and the InterPro Search tool [47]. Lastly, all multiple sequence alignments were generated by Clustal Omega using the EBI web service [48].

### 2.2. Homology-Based Modeling and 3D-Structure Validation

The three-dimensional (3D) structures of the putative functional domains, RBD and PIKKc, detected in both TOR-like amoebic proteins, were predicted using the I-TASSER suite, a platform for automatic homology-based modeling [49,50,51]. The resulting models were improved using two on-site algorithms for atomic-level protein structure refinement: ModRefiner [52] and FG-MD [53]. The all-atom structure accuracy of the top-ranked 3D models was validated using the MolProbity online service [54]. The protein structures were analyzed using the interactive molecular graphics system offered by PyMol (Version 2.0; Schrödinger, LLC.) and UCSF Chimera [55].

### 2.3. In Silico Analysis of the Rapamycin-Binding Site

The ligand-binding site and the putative rapamycin-interacting residues were determined using the IntFOLD suite [56,57]. The RBD polypeptide sequences from *Eh*FRAP (2002–2105 residues) and *Eh*TOR2 (1841–1944 residues) were the data used to predict the protein-ligand interactions with FunFOLD [58,59], using the default settings. The ligand-binding site accuracy was validated using FunFOLDQA [60]. The protein-ligand 3D structures were analyzed using the PyMol System and the BIOVIA Discovery Studio Visualizer (v21.1.0.20298, Dassault Systèmes), along with the PLIP web service [61].

### 2.4. Computational Prediction of Protein-Protein Interactions

The protein-protein interacting partners of both TOR-like amoebic proteins were detected using the STRING server [62]. The predicted protein-protein interactions’ (PPI) scores of >0.9 were considered significant. All predicted interacting partners were also analyzed using the InterPro Search engine [47].

## 3. Results

### 3.1. Entamoeba histolytica Contains Two Genes Encoding TOR-like Proteins

The keyword search within the AmoebaDB repository returned two genes encoding TOR-like proteins (*E. histolytica* HM-1:IMSS genome database), annotated as putative FKBP-rapamycin associated protein (FRAP), from now on referred to as *Eh*FRAP (291.7 kDa, encoded by EHI_155160), and putative phosphatidylinositol 3-kinase Tor 2, from now on referred to as *Eh*TOR2 (269.5 kDa, encoded by EHI_169320). Table 1 summarizes the physicochemical properties of these proteins.

### 3.2. TOR-like Amoebic Proteins Show the Canonical Domain Organization

**Figure 2:**
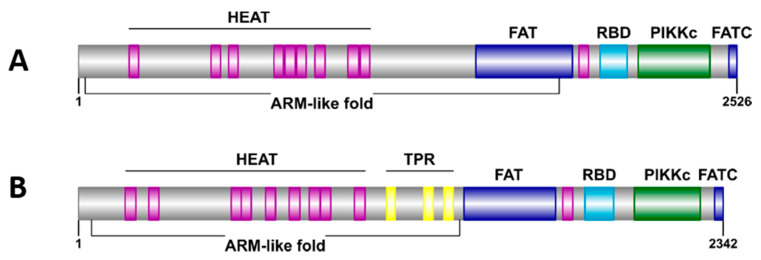
Schematic representation of the *Eh*FRAP (**A**) and *Eh*TOR2 (**B**) domain organization. Protein features were identified by primary structure analysis. While the tetratricopeptide repeats (TPR) are in yellow, the colors for HEAT, FAT and FATC, RBD, and PIKKc were as in Figure 1. Drawings generated with the IBS program.

As expected, the combined outcome of the four computational tools (CD-Search, Pfam, REP2, and InterPro) confirmed that both TOR-like amoebic proteins display the canonical domain organization: HEAT-FAT-RBD-PIKKc-FATC (Figure 2). Furthermore, the InterPro results revealed that each N-terminal HEAT-containing region (residues: 25–1484 for *Eh*FRAP and 46–1285 for *Eh*TOR2) shows an armadillo-like folding, a common structural feature of protein domains that include HEAT repeats [63].

A comparative analysis by DELTA-BLAST (domain enhanced lookup time accelerated BLAST) provided additional data about primary sequence homologies among the amoebic, yeast, and human TOR-like proteins (Table 2). As expected, both yeast TOR proteins share high levels of identity and similarity (68% and 82%), while mTOR shares moderate homology with them (40–42% identity; 58–60% similarity). With this reference, it seems reasonable to suggest that both TOR-like amoebic proteins share moderate homology with their yeast and human counterparts, showing identities of 29–33% and similarities of 47–52%. Furthermore, unlike yeast TOR proteins, the amoebic homologs share moderate identity and similarity (38% and 56%, respectively).

### 3.3. TOR-like Amoebic Proteins Have Conserved RBD and PIKKc Domains

The multiple sequence alignments of the RBD and PIKKc domains, performed with Clustal Omega, generated the initial knowledge about the structural conservation of the functional residues of TOR-like amoebic proteins. Overall, amoebic domains share significant homology with their counterparts from yeast and humans.

**Figure 3:**
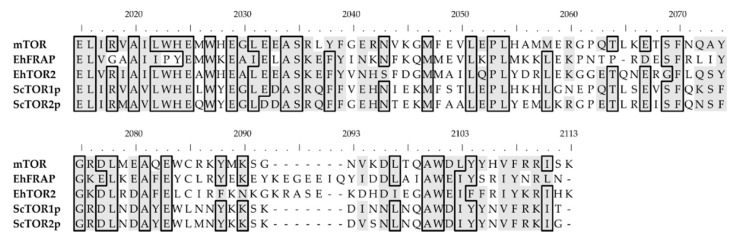
Multiple sequence alignment of rapamycin-binding domains. Reference sequence (range): mTOR (2015–113), *Eh*FRAP (2002–2105), *Eh*TOR2 (1841–1944), *Sc*TOR1p (1952–2049), and *Sc*TOR2p (1955–2052). Identical residues are within boxes, while those which are similar are shaded (gray). The top-ruler residue-numbering corresponds to the human mTOR sequence.

While *Eh*FRAP and *Eh*TOR2 show 46% identical RBD sequences, these protein domains share 31–40% identity compared to their yeast and human counterparts (Figure 3). Furthermore, both amoebic sequences conserve most residues that are presumed essential for molecular interaction with the FKBP-rapamycin complex [6,64], as identified for the human mTOR counterpart: W2027, L2031, S2035, Y2038, F2039, T2098, W2101, D2102, Y2105, and F2108.

**Figure 4:**
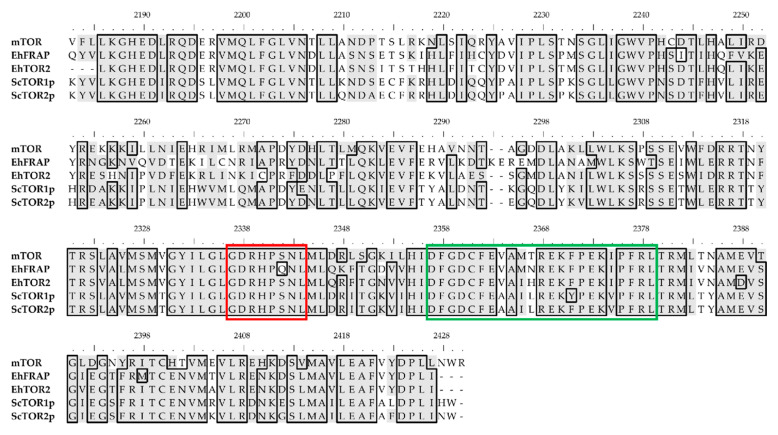
Multiple sequence alignment of PIKKc domains. Reference sequence (range): mTOR (2183–2430), *Eh*FRAP (2176–2422), *Eh*TOR2 (2019–2260), *Sc*TOR1p (2120–2366), and *Sc*TOR2p (2124–2370). The sequence of functional loops is within colored boxes: catalytic (red) and activation (green). Similar/identical residues, reference sequences, and top-ruler numbers are as described in Figure 3.

Remarkably, TOR-like amoebic proteins contain highly conserved PIKKc domains (81% identical), which share significant identity (65–66%) with their yeast and human counterparts (Figure 4). Furthermore, the putative catalytic loop of both amoebic PIKKc domains includes three conserved residues, identified in human mTOR as critical for kinase function: D2338 (D2333 in *Eh*FRAP; D2171 in *Eh*TOR2), which plays a significant role in the orientation/activation of the substrate hydroxyl group for nucleophilic attack; H2340 (H2335 in *Eh*FRAP; H2173 in *Eh*TOR2), which participates in stabilizing the buildup of charge at the transition state; and N2343 (N2338 in *Eh*FRAP; N2176 in *Eh*TOR2), which serves as a metal-ligand. A notable finding was that the amoebic PIKKc domains show a highly conserved activation loop, sharing 85–90% identity with their yeast and human homologs. Interestingly, the mTOR activation loop is essential for both the function and regulation of kinase activity [65].

The homology-based 3D models of the RBD and PIKKc domains provided additional information about the structure-function relationship of the TOR-like amoebic proteins. As a general process, the five top-ranked 3D structures, generated automatically by I-TASSER, were improved using two methods: ModRefiner and FG-MD. Ramachandran plots and MolProbity analyses validated the quality of the refined 3D models: residues in favored regions were ≥85%, and in allowed regions were ≥95% (Supplementary Figure S1A,C), and clashscore value and MolProbity scores in the ≥66% percentile.

**Figure 5:**
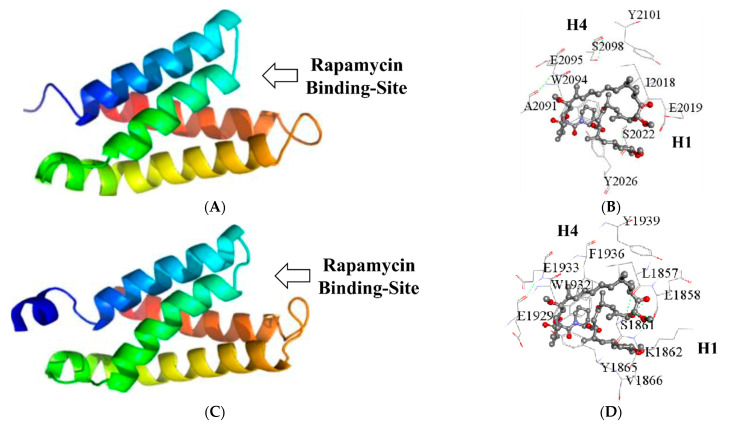
Predicted tertiary structure for the rapamycin-binding domain (RBD) of both TOR-like amoebic proteins. Best 3D model (ribbon representation) for the RBD of *Eh*FRAP (**A**) and *Eh*TOR2 (**C**). Rainbow-colored from amino (blue) to carboxy (red). An arrow indicates the respective predicted rapamycin binding-site. Best models for the ligand-binding cleft and residues interacting with rapamycin: *Eh*FRAP (**B**) and *Eh*TOR2 (**D**). H1 and H4 show the relative locations of the respective α-helix 1 and α-helix 4. Rapamycin (balls and sticks) is colored by element using the default settings.

As suspected, the best 3D model for the RBD of each amoebic protein (*Eh*FRAP and *Eh*TOR2) showed the typical folding pattern: a four-helix bundle with the amino and carboxy termini close to each other (Figure 5). Furthermore, supplementary analysis by 3D-structure superposition confirmed their similarity to functional counterparts (Supplementary Figure S1B,D).

It is worth noticing that the first and fourth helix (H1 and H4) formed a cleft near their cross-section, resembling the rapamycin-binding site of the RBD of human mTOR [6,64]. The prediction of the binding site and ligand-protein interactions by IntFold provided further insights about the latter (Figure 5B,D). As expected, both ligand-binding sites, *Eh*FRAP RBD (consisting of residues I2018, E2019, S2022, and Y2026 from H1, and A2091, W2094, E2095, S2098, and Y2101 from H4) and *Eh*TOR2 RBD (involving residues L1857, E1858, S1861, K1862, Y1865, and V1866 of H1, and E1929, W1932, E1933, F1936, and Y1939 of H4), showed potential binding to rapamycin through a significant number (around ten) of non-covalent interactions (Supplementary Table S1).

**Figure 6:**
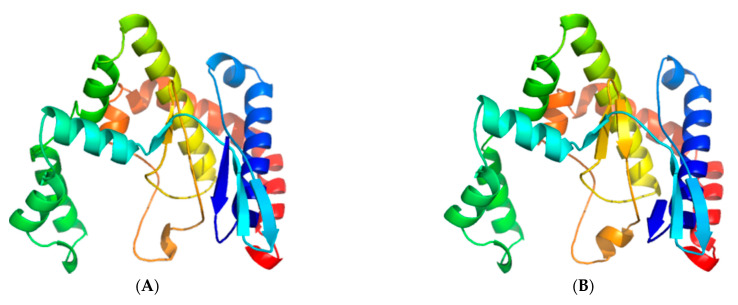
Predicted tertiary structure for the kinase domain (PIKKc) of both TOR-like amoebic proteins. Best 3D model (ribbon representation) for the PIKKc: *Eh*FRAP (**A**) and *Eh*TOR2 (**B**). Rainbow-colored from amino (blue) to carboxy (red).

Similarly, the best 3D model for the PIKKc domain of each TOR-like amoebic protein: *Eh*FRAP and *Eh*TOR2, displayed a remarkably conserved folding pattern (Figure 6). Furthermore, 3D-structure superpositions confirmed their high similarity level to functional kinase domains (Supplementary Figure S2).

### 3.4. TOR-like Amoebic Proteins Are Potential Participants in PPI Networks

An analysis of the predicted protein-protein interaction networks for *Eh*FRAP and *Eh*TOR2 provided additional information on the protein structure-function relationships and their ability to bind/interact with amoebic TORC components or any other cellular participants involved in TOR-associated signaling pathways. STRING analysis returned ten high confidence potential PPI partners for each TOR-like amoebic protein, with nine being common to both (Table 3). Of these, two proteins, EHI_040260 (HEAT repeat domain-containing protein) and EHI_098410 (WD domain-containing protein), belong to the Raptor (regulatory associated protein of TOR) family, whose members are TORC1 components. Another protein, EHI_081760 (cytosolic regulator pianissimo), shows sequence similarity to Rictor (rapamycin-insensitive companion of TOR), a TORC2 component. Furthermore, two proteins, EHI_178770 and EHI_023210 (GTP-binding proteins, putative), belong to the Gtr1/RagA family (small GTPases of Ras superfamily), whose members are important mediators of amino acid signaling to TORC1. A supplementary search within the AmoebaDB repository revealed an Lst8-like protein, EHI_202590, sharing structural similarity with functional WD repeat LST8 family members, which are essential components of both complexes, TORC1 and TORC2.

### 3.5. TORC1 and TORC2 in E. histolytica: In Silico Characterization

A complementary biocomputational analysis of the putative core components of TORC1 and TORC2 provided further insights into the structural conformation of these complexes in *E. histolytica* (Table 4). As expected, either *Eh*FRAP or *Eh*TOR2 can form the *Eh*TORC1 complex through PPI with a Kog1p/Raptor homolog: *Eh*Raptor-1 (EHI_040260) or *Eh*Raptor-2 (EHI_098410), and the *Eh*Lst8 protein (EHI_202590). Remarkably, these components showed significant similarity to their functional counterparts. For instance, *Eh*Raptor proteins are 48–59% similar to the yeast Kog1p (SGD: YHR186C) and human Raptor (NCBI: NP_065812), whereas *Eh*Lst8 is 57–59% similar to the yeast Lst8p (SGD: YNL006W) and human LST8 (NP_001186102). Furthermore, as suspected, *Eh*FRAP or *Eh*TOR2 can also form the *Eh*TORC2 complex through PPI with *Eh*Lst8 and the Avo3p/Rictor homolog, *Eh*Rictor (EHI_081760). Moreover, *Eh*Rictor showed significant similarity (50–56%) to the yeast Avo3p (SGD: YER093C) and human Rictor (NCBI: NP_689969).

Interestingly, it seems that both amoebic complexes, *Eh*TORC1 and *Eh*TORC2, have minimal core proteins for TORC functions. However, the apparent lack of other components found in yeast and human TORC protein complexes allowed us to speculate on the existence of *Eh*TORC-specific components not detectable by typical homology-based biocomputational analyses. In this regard, the biochemical analysis of the amoebic complexes will provide further knowledge about their functional composition.

## 4. Discussion

Using a biocomputational approach, we performed an in silico characterization of two annotated TOR-like amoebic proteins: *Eh*FRAP and *Eh*TOR2. As expected, both showed the canonical domain organization, including a long N-terminal region that shows an armadillo-like fold (formed by tandem HEAT repeats [66]), which is important for mediating protein-protein interactions [46,67,68]. Conversely, their C-terminal regions showed a typical arrangement consisting of two putative functional domains, RBD and PIKKc, flanked by the FAT and FATC domains. Interestingly, this coexistence suggests a FAT-FATC structural conformation that ensures kinase function. It is also likely that the FAT domains could function as structural scaffolds or protein-binding domains [69,70].

Regarding the rapamycin-binding domain (RBD), both TOR-like amoebic proteins contain conserved residues critical for ligand-binding, located at the H1 and H4 α-helices, predicting a rapamycin-binding cleft structurally similar to that of mTOR RBD [6,71]. Furthermore, a supplementary analysis of the folding pattern and its ability to interact with the ligands (i.e., rapamycin), based on structural comparisons with functional homologs, confirmed the latter. Likewise, the kinase domain (PIKKc) of both TOR-like amoebic proteins contain conserved residues that form two structural features, the catalytic and activation loops, which are normally associated with protein-kinase function and regulation [72,73]. Moreover, both catalytic loops include the Asp-His-Asn triad, which in the human mTOR PIKKc is directly involved in kinase activity [65]. Their predicted folding pattern also showed consistent structural similarity with functional homologs. Altogether, these findings suggest that *Eh*FRAP and *Eh*TOR2 have favorable structural features to function as rapamycin-binding proteins with kinase activity.

Further biocomputational analyses revealed that both TOR-like amoebic proteins have structural features that allow them to participate in protein-protein interaction (PPI) networks. Moreover, the gene ontology examinations of the putative *Eh*FRAP/*Eh*TOR2 interaction partners showed that both networks include proteins sharing similarities with functional homologs involved in TORC-associated signaling pathways. So far, these results confirm that *E. histolytica* encodes TOR-interacting protein homologs, which could bind to *Eh*FRAP or *Eh*TOR2 and form TORC-like amoebic complexes.

As a final thought, it is reasonable to assume that our study paves the way for future research on biochemical and cellular processes associated with TOR/TORC functions in *E. histolytica*. However, considering that our computational approach is just the first step, we recognize that experimental validation (by in vivo and in vitro approaches) is mandatory before directing efforts to discover new or improved drugs against amoebiasis. In this regard, biochemical studies aimed at characterizing the protein kinase activity or determining the ligand-binding properties represent a feasible starting point [11,74,75,76]. Furthermore, the RNA interference and downregulation of gene expression appears to be a reliable approach to determine the precise function of *Eh*FRAP and *Eh*TOR2, given that gene disruption is not feasible for *E. histolytica* by standard molecular genetics methods [77,78]. Alternatively, the heterologous complementation of yeast TOR *null*-mutants remains viable as a traditional approach to assess gene function [79,80,81].

## 5. Conclusions

Our in silico findings show apparent internal reliability regarding the predicted structure-function relationship for both TOR-like amoebic proteins, providing a solid foundation for further studies. They also suggest that *Eh*FRAP and *Eh*TOR2 are promising targets for specific inhibition, leading to the disruption of TOR-associated signaling pathways in *E. histolytica*. Overall, this study represents the first approach to establish their functional role in the protozoan pathobiology and, consequently, their potential as targets for the therapeutic control of human amoebiasis.

## Figures and Tables

**Table 1:** Physicochemical properties of the TOR-like amoebic proteins.

Parameter	*Eh*FRAP	*Eh*TOR2
Amino acid residues	2526	2342
Predicted molecular mass (kDa)	291.7	269.5
Theoretical pI	6.35	5.73
Asp + Glu	363	309
Arg + Lys	347	266
Instability index	42.14	42.73
Aliphatic index	95.54	104.18
Grand Average of Hydropathicity	−0.357	−0.174

**Table 2:** Pairwise comparisons of TOR-like amoebic proteins with yeast and mammalian counterparts. Percentage values of identity and similarity (global alignments).

	Identity				
	*Eh*FRAP	*Eh*TOR2	*Sc*TOR1p	*Sc*TOR2p	mTOR
*Eh*FRAP	- -	38	30	29	32
*Eh*TOR2	56	- -	33	33	33
*Sc*TOR1p	48	52	- -	68	40
*Sc*TOR2p	47	52	82	- -	42
mTOR	50	51	58	60	- -
	**Similarity**				

NCBI Acc. No. (Ref. Seq.): XP_650639 (*Eh*FRAP), XP_651206 (*Eh*TOR2), NP_012600 (*Sc*TOR1p), NP_012719 (*Sc*TOR2p), NP_001373429 (mTOR). “- -” means not compared due to full matching.

**Table 3:** PPI networks predicted by STRING for *Eh*FRAP and *Eh*TOR2.

Identifier	Protein Annotation	Interaction Score ^1^	
		*Eh*FRAP	*Eh*TOR2
EHI_040260	HEAT repeat domain-containing protein	0.993	0.998
EHI_098410	WD domain-containing protein	0.993	0.993
EHI_081760	Cytosolic regulator pianissimo, putative	0.973	0.973
EHI_168210	Protein kinase domain-containing protein	0.953	0.953
EHI_178770	GTP-binding protein, putative	0.950	0.950
EHI_000740	Uncharacterized protein, PP2A-binding	NP	0.949
EHI_093770	Bromodomain protein, putative	0.945	0.945
EHI_023210	GTP-binding protein, putative	0.942	0.942
EHI_184240	PI3K/PI4K domain-containing protein	0.932	0.932
EHI_004790	Ser/Thr protein kinase, putative	0.927	0.927
EHI_044470	Non-specific Ser/Thr protein kinase	0.927	NP

^1^ Network statistics: clustering coefficient is 0.79; PPI enrichment *p*-value < 10^−5^. The interaction score threshold was 0.7 (high confidence) for both analyses. NP, no PPI predicted.

**Table 4:** Components of *S. cerevisiae* TORC1 and TORC2 and their homologs in humans and amoeba.

*S. cerevisiae*	*H. sapiens*	*E. histolytica*
TORC1		
TOR1p or TOR2p	mTOR	*Eh*FRAP or *Eh*TOR2
Kog1p	Raptor	*Eh*Raptor-[1/2] (EHI_040260/EHI_098410)
Lst8p	mLST8	*Eh*Lst8 (EHI_202590)
Tco89p	-	-
TORC2		
TOR2p	mTOR	*Eh*FRAP or *Eh*TOR2
Avo1p	mSIN1	-
Avo2p	-	-
Avo3p	Rictor	*Eh*Rictor (EHI_081760)
Lst8p	mLST8	EhLst8 (EHI_202590)
Bit61p	-	-

## Data Availability

All data presented in this study are available on request from the corresponding author, without undue reservation, to any qualified researcher.

## Supplementary Materials

The following are available online at https://www.mdpi.com/article/10.3390/genes12081139/s1, Figure S1: Analysis of the predicted tertiary structure for the rapamycin-binding domain (RBD) of both TOR-like amoebic proteins, Figure S2: Analysis of the predicted tertiary structure for the kinase domain (PIKKc) of both TOR-like amoebic proteins, Table S1: Comparison of rapamycin-interacting residues of human mTOR in contrast to those predicted for the amoebic homologs.
