# Supplementary material for: Bioinformatic Analysis of Two TOR (Target of Rapamycin)-Like Proteins Encoded by Entamoeba histolytica Revealed Structural Similarities with Functional Homologs
Source: Genes (Basel). 2021 Jul 28;12(8):1139. doi: 10.3390/genes12081139 (PMC8391992; doi:10.3390/genes12081139)
Supplement: Supplementary file 1 [file genes-12-01139-s001.zip › genes-1302312-supplementary.pdf]

Genes

Supplementary Material

Bioinformatic Analysis of Two TOR (Target of Rapamycin)-Like Proteins Encoded by *Entamoeba histolytica* Revealed Structural Similarities with Functional Homologs

Patricia L.A. Muñoz-Muñoz, Rosa E. Mares-Alejandre, Samuel G. Meléndez-López and Marco A. Ramos-Ibarra*

Biotechnology and Biosciences Research Group, Faculty of Chemical Sciences and Engineering, Autonomous University of Baja California, Tijuana, BCN, Mexico. * Correspondence: mramos@uabc.edu.mx.

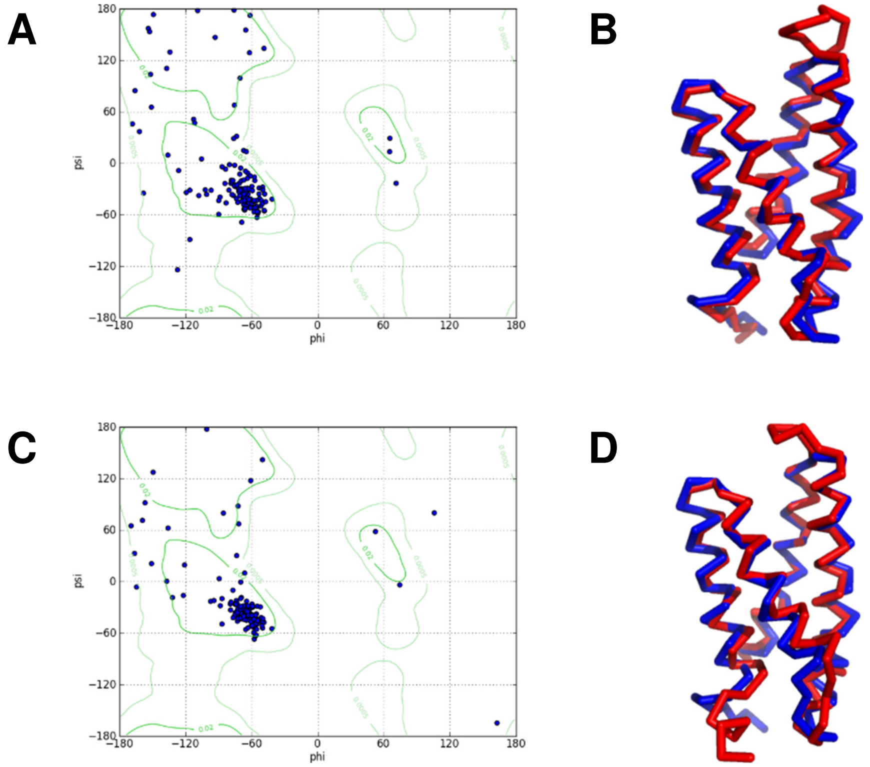

Figure S1. Analysis of the predicted tertiary structure for the rapamycin-binding domain (RBD) of both TOR-like amoebic proteins. Respective Ramachandran plots and 3D-structure superpositions (template colored in blue, RBD of the human mTOR): *Eh*FRAP (**A** and **B**) and *Eh*TOR2 (**C** and **D**).

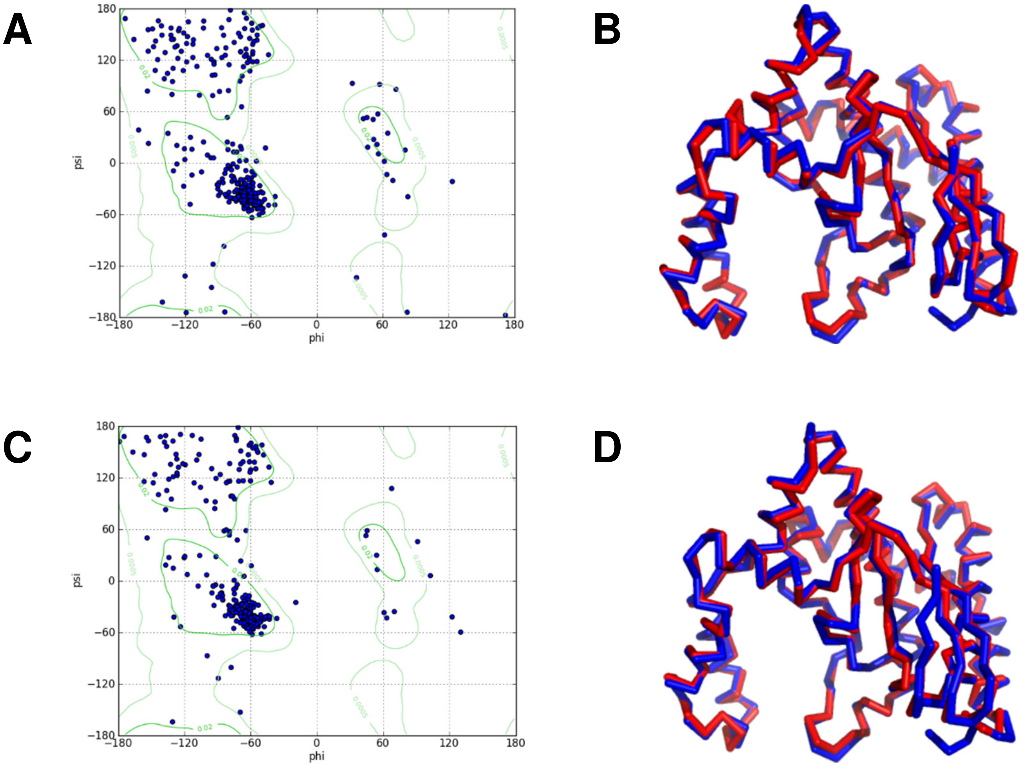

Figure S2. Analysis of the predicted tertiary structure for the kinase domain (PIKKc) of both TOR-like amoebic proteins. Respective Ramachandran plots and 3D-structure superpositions (template colored in blue, PIKKc of the human mTOR): *Eh*FRAP (**A** and **B**) and *Eh*TOR2 (**C** and **D**).

Table S1. Comparison of rapamycin-interacting residues of human mTOR in contrast to those predicted for the amoebic homologs.

**mTOR**	***Eh*FRAP**	***Eh*TOR2**
L2031	I2018	L1857
NR	E2019	E1858
S2035	S2022	S1861
NR	NP	K1862
Y2038	NP	NP
F2039	Y2026	Y1865
NR	NP	V1866
T2098	A2091	E1929
W2101	W2094	W1932
D2102	E2095	E1933
Y2105	S2098	F1936
F2108	Y2101	Y1939

NR, not reported. NP, not predicted.
